# The *Anopheles coluzzii* microbiome and its interaction with the intracellular parasite *Wolbachia*

**DOI:** 10.1038/s41598-020-70745-0

**Published:** 2020-08-14

**Authors:** Timothy J. Straub, W. Robert Shaw, Perrine Marcenac, Simon P. Sawadogo, Roch K. Dabiré, Abdoulaye Diabaté, Flaminia Catteruccia, Daniel E. Neafsey

**Affiliations:** 1 Infectious Disease and Microbiome Program, Broad Institute of MIT and Harvard, Cambridge, MA 02144 USA; 2 Department of Immunology and Infectious Diseases, Harvard T.H. Chan School of Public Health, Boston, MA 02115 USA; 3 Institut de Recherche en Sciences de La Santé/Centre Muraz, O1 BP 390, Bobo-Dioulasso 01, Burkina Faso

**Keywords:** Metagenomics, Microbiome, Malaria, Entomology

## Abstract

*Wolbachia*, an endosymbiotic alpha-proteobacterium commonly found in insects, can inhibit the transmission of human pathogens by mosquitoes. Biocontrol programs are underway using *Aedes aegypti* mosquitoes trans-infected with a non-natural *Wolbachia* strain to reduce dengue virus transmission. Less is known about the impact of *Wolbachia* on the biology and vectorial capacity of *Anopheles* mosquitoes, the vectors of malaria parasites. A naturally occurring strain of *Wolbachia*, *w*Anga, infects populations of the major malaria vectors *Anopheles gambiae* and *Anopheles coluzzii* in Burkina Faso. Previous studies found *w*Anga infection was negatively correlated with *Plasmodium* infection in the mosquito and *w*Anga influenced mosquito egg-laying behavior. Here, we investigate *w*Anga in natural populations of *An. coluzzii* and its interactions with other resident microbiota using targeted 16S sequencing. Though we find no major differences in microbiota composition associated with *w*Anga infection, we do find several taxa that correlate with the presence or absence of *w*Anga in female mosquitoes following oviposition, with the caveat that we could not rule out batch effects due to the unanticipated impact of *w*Anga on oviposition timing. These data suggest *w*Anga may influence or interact with the *Anopheles* microbiota, which may contribute to the impact of *w*Anga on *Anopheles* biology and vectorial capacity.

## Introduction

Despite progress in control efforts over the past decade, malaria remains a major global health problem, with over 200 million reported cases each year^1^. The most lethal form of malaria is caused by the parasite *Plasmodium falciparum*, which remains endemic across much of sub-Saharan Africa. Vector control has been the most important contributor to reduction in global mortality and morbidity^2–6^. A recent stall in the decline of malaria^1,6^, and the increasingly widespread observations of insecticide resistance in mosquito populations^7^, however, highlight the need for novel approaches to malaria vector control.

Manipulation of mosquito microbiota, and in particular the introduction of the alphaproteobacterial endosymbiont *Wolbachia*, is an example of a promising new avenue of vector control^8,9^. *Wolbachia* infection reduces the capacity of *Aedes* mosquitoes to transmit dengue virus and other arboviruses through mechanisms that are incompletely understood, but which may include manipulation of the mosquito innate immune response, nutrient competition, and life-shortening of the mosquito^10–12^. In *Ae. aegypti*, transinfected *Wolbachia* has been shown to significantly alter the mosquito microbiome, suggesting the existence of complex interactions between resident microbes that may also influence vectorial capacity^13^. *Wolbachia* can be vertically transmitted from mother to offspring through infection of the germ line, thus allowing it to persist across generations once introduced. In *Aedes* and many other arthropod hosts, the spread of *Wolbachia* is enhanced through a phenomenon termed cytoplasmic incompatibility (CI), in which uninfected females that mate with infected males produce sterile broods, giving a strong reproductive advantage to infected females^4,14^.

*Wolbachia* has recently been detected with PCR-based and whole-genome sequencing approaches in several important African malaria vector species, *Anopheles coluzzii*, *Anopheles gambiae*, and *An. arabiensis*^15–21^. These findings, although called into question by a recent study^22^, have challenged the notion that anopheline mosquitoes do not harbor natural *Wolbachia* infections. The *Wolbachia* strain identified in Burkina Faso, named *w*Anga, has been found to exhibit very low infection intensity in *An. gambiae* and *An. coluzzii*^15,16^. Moreover, *w*Anga is present at low to intermediate frequencies in wild populations, most likely because it does not induce CI^16^. *Wolbachia* infections are negatively correlated with *P. falciparum* in *An. coluzzii*^16,19^, raising interest in understanding the mechanism by which these bacteria affect the capacity of *Anopheles* mosquitoes to transmit malaria parasites.

To aid in understanding the biological effects of *w*Anga infection on *An. coluzzii*, and the potential avenues by which it could be impacting malaria vectorial capacity, we collected blood-fed adult female mosquitoes in Burkina Faso. Using 16S-based assays on DNA extracted from mosquito carcasses, we found that while there are no major differences in the microbiome composition between mosquitoes with vs. without *w*Anga, certain bacterial taxa appear to be positively or negatively associated with this *Wolbachia* strain. This could suggest that some residents of the *Anopheles* microbiota may promote the ability of *w*Anga to colonize the mosquito host, while others may disrupt it. Interpretation of these associations between *w*Anga and other microbes is complicated by the biological effects of *w*Anga infection on *An. coluzzii* oviposition timing^16^, motivating further studies to explore the interaction of these effects on microbiome profile.

## Results

### The *An. coluzzii microbiota*

**Figure 1:**
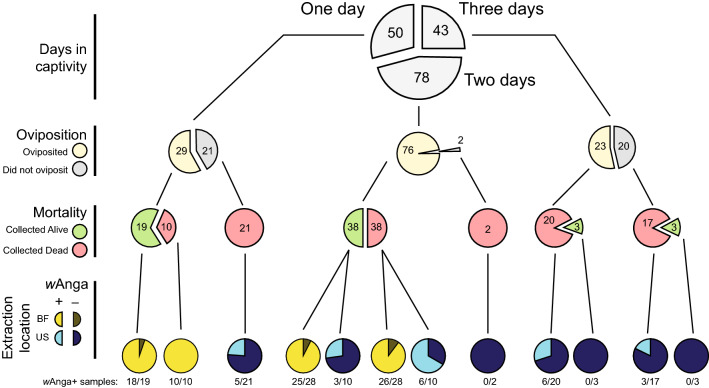
Breakdown of samples collected. A total of 171 samples were captured and then collected for DNA extraction after up to 3 days in captivity (top row, numbers indicate samples collected on each given day). Mosquitoes were collected for extraction after oviposition (second row, in beige), upon death in captivity regardless of oviposition (mortality, third row, in red), or at the end of 3 days in captivity regardless of oviposition or death. Due to study design, virtually all samples collected early (i.e., one and 2 days in captivity) underwent DNA extraction in Burkina Faso (BF, yellow circles), while the rest were extracted at a later date in the United States (US, blue circles). *w*Anga infection status was performed using a nested PCR, with the infection rate signified by the size of the bright blue and yellow arcs in the last row. The fraction of *w*Anga-positive samples for each bottom pie chart are specified by the numbers in the bottom row. Due to the accelerated oviposition impact of *w*Anga, *w*Anga infection correlated with days in captivity (p = 8.4 × 10^–9^), oviposition (p = 7.2 × 10^–10^), whether the mosquito was collected still alive or dead (p = 0.0094), and DNA extraction location (p < 10^–15^). p-values reported are from Fisher’s Exact Tests and have been corrected for multiple hypothesis testing using Benjamini-Hochberg.

We analysed 171 mosquitoes (Fig. 1), 102 of which were determined to be infected with wAnga by 16S PCR^16^, by targeted 16S rRNA sequencing and obtained a mean of 72,656 reads per sample (Supplementary Fig. 1a); 144 samples exhibited at least 10,000 reads and were retained for further analysis. We observed a total of 3,189 operational taxonomic units (OTUs) at 97% identity; filtering to remove low abundance and rare OTUs (defined by those seen in only one sample or fewer than 50 reads across all samples) reduced this number to 916 OTUs. (Supplementary Fig. 1a). Only four OTUs were “core” (i.e. present in every one of the 144 mosquitoes), three of which were assigned to *Acinetobacter* and one to *Comamonadaceae*, all of which were within Proteobacteria. These core OTUs ranged in relative abundance within individual mosquitoes, sometimes as high as 80% of all bacteria (Supplementary Fig. 1b). Alpha rarefaction analyses indicated that we sequenced to a sufficient depth such that we are accurately estimating the diversity of the microbiota of the mosquitoes, and that the presence or absence of a given OTU is not likely due to insufficient sequencing depth (Supplementary Fig. 1c).

**Figure 2:**
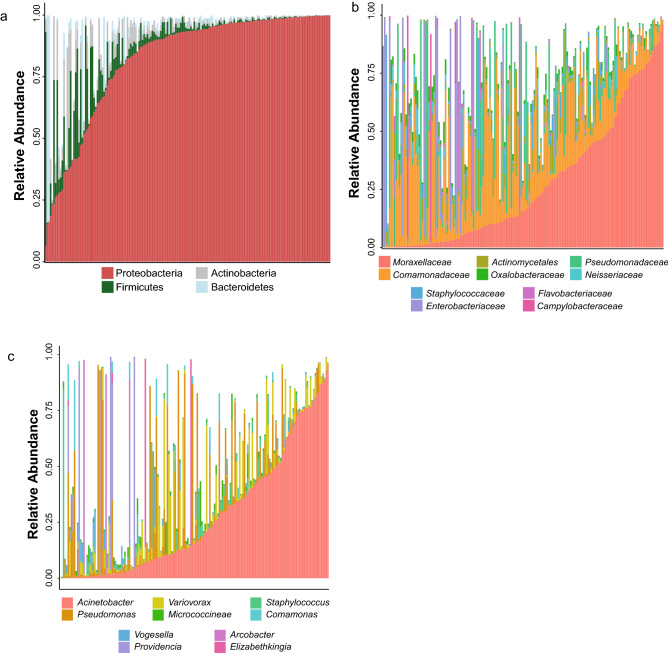
The bacterial composition of the *An. coluzzii* microbiota. Per sample distributions of phyla (**a**), families (**b**), and genera (**c**) are shown. X axis represents individual mosquitoes and Y axis indicates the relative abundance of each taxon, which are colour-coded. For each, samples have been sorted on the abundance of the most dominant taxon. Only the most abundant taxa are displayed for clarity. The microbiome is dominated by Proteobacteria in the majority of mosquitoes analysed, though there was substantial variation between individuals. At the family and genus level, there is increased diversity, though in many of the samples *Moraxellaceae* and the genus *Acinetobacter* were dominant. These results are consistent with previously published reports on *Anopheles*.

Overall, our study confirmed previous reports showing that the *An. coluzzii* microbiome is diverse and composed of many phyla^15,23–26^. It is dominated by Proteobacteria, while Firmicutes, Actinobacteria, and Bacteroidetes are abundant secondary phyla (Fig. 2a). Greater diversity is apparent at the family level. Mosquito microbiomes were dominated by bacteria from *Comamonadaceae*, *Moraxellaceae*, *Pseudomonadaceae*, and occasionally *Enterobacteriaceae* (Fig. 2b). At the genus level, we observed *Acinetobacter*, *Pseudomonas*, and *Variovorax* as the most abundant genera (Fig. 2c), consistent with core OTU-level analysis (Supplementary Fig. 1b) and also detected *Comamonas* and *Elizabethkingia*. We observed significant variation among individual mosquitoes at each of these levels of taxonomy, again consistent with previous reports that indicated individual mosquito microbial compositions can vary widely^24,25,27–30^. To note, we were able to detect one OTU assigned to *Wolbachia*, which was observed in only a single mosquito sample (42 reads; 0.04% relative abundance).

### *w*Anga infection has limited effects on overall microbiota composition

**Figure 3:**
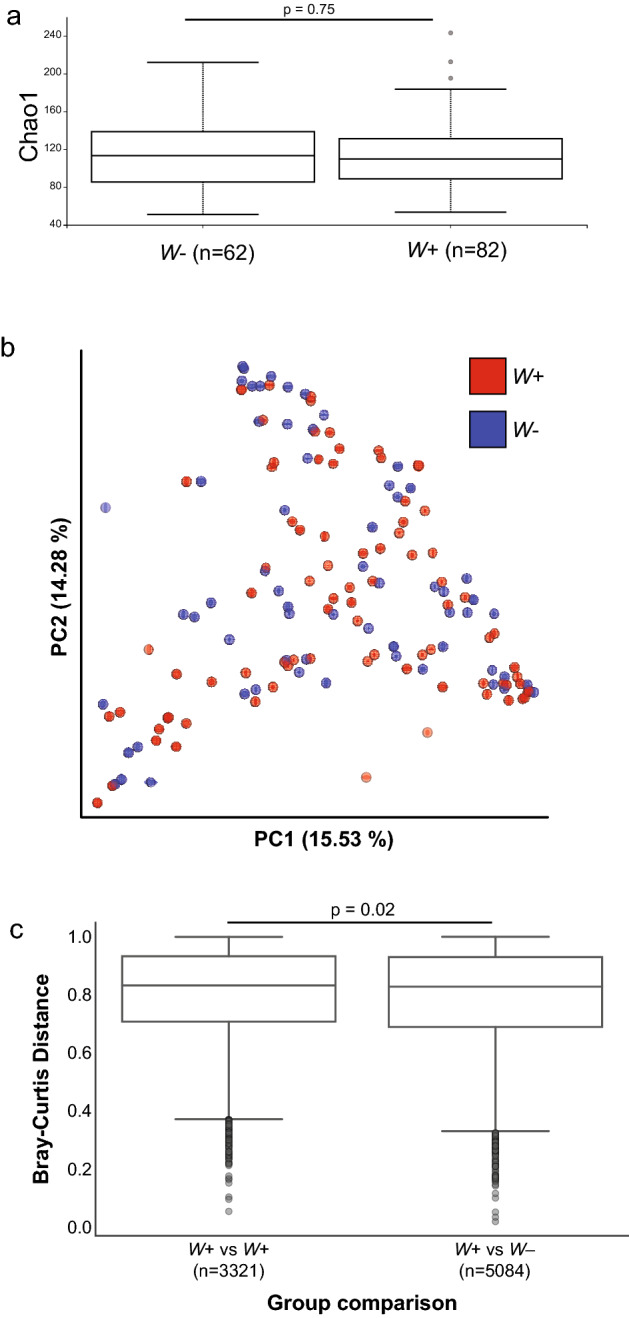
*w*Anga infection minimally alters overall composition of microbiota. Whether a mosquito was infected with *w*Anga did not alter the overall composition of the microbiota. Alpha diversity measurements using the Chao1 metric were not significantly different between infected and uninfected mosquitoes (**a**). Principal Coordinate Analysis indicates no clear visual distinction or clustering between *w*Anga infected (red) and uninfected (blue) mosquitoes (**b**). There was a very slight, but significant (PERMANOVA pseudo-F = 1.99, p = 0.023) increase in Bray–Curtis distances between uninfected and infected mosquitoes than distances within individual infected mosquitoes, indicating that infected mosquitoes are more similar to each other in composition than to uninfected mosquitoes (**c**). The reciprocal measure, in which uninfected mosquitoes were more similar to uninfected than infected mosquitoes, was also true (data not shown).

We next explored the relationship between several sample features (see Fig. 1) and microbiota diversity. Alpha diversity, as measured by the metric Chao1, indicating the richness of the bacterial community, did not differ significantly between mosquitoes with respect to *w*Anga infection status (Kruskal–Wallis; H = 0.1, p = 0.75) (Fig. 3a). In addition, Chao1 values did not differ significantly according to whether they were alive or dead at time of collection for DNA extraction (referred to as ‘mortality’ here) (Kruskal–Wallis, p > 0.5) (Supplementary Fig. 2a). The location in which the sample was extracted (either Burkina Faso or our laboratory in Boston, USA) also had no significant effect on Chao1 values (Kruskal–Wallis, p > 0.5) (Supplementary Fig. 2b). However, the day mosquitoes were collected had a significant effect on alpha diversity (Kruskal–Wallis, H = 19.8, p = 5 × 10^–5^), with mosquitoes collected on the first day of collection exhibiting significantly more diversity than those collected on the second and third days (Kruskal–Wallis, p < 10^–4^) (Supplementary Fig. 2c). Notably, the sample features ‘*w*Anga infection status’, ‘mortality’, and ‘DNA extraction location’ were also correlated with each other to varying degrees (Fig. 1; Supplementary Fig. 3).

There was no clear visual clustering by Principal Coordinate Analysis of Bray–Curtis distances (i.e., beta diversity) of samples for the measured sample attributes, including *w*Anga infection status (Fig. 3b), indicating that neither technical nor biological factors appeared to affect the overall composition of the microbiome. We also tested for an association between alpha diversity and other variables: *w*Anga infection status, extraction location, and collection day. All three associations were significant by PERMANOVA of Bray–Curtis distances (corrected p < 0.05, 10,000 permutations), indicating that despite the absence of a clear visual pattern, samples from the same extraction batch, infection status and collection day were significantly more similar to each other (Fig. 3c). As all three variables were correlated to each other (Fig. 1; Supplementary Fig. 3), it is difficult to infer the source of causality for these observations.

### Interactions between *Wolbachia* and individual OTUs

**Figure 4:**
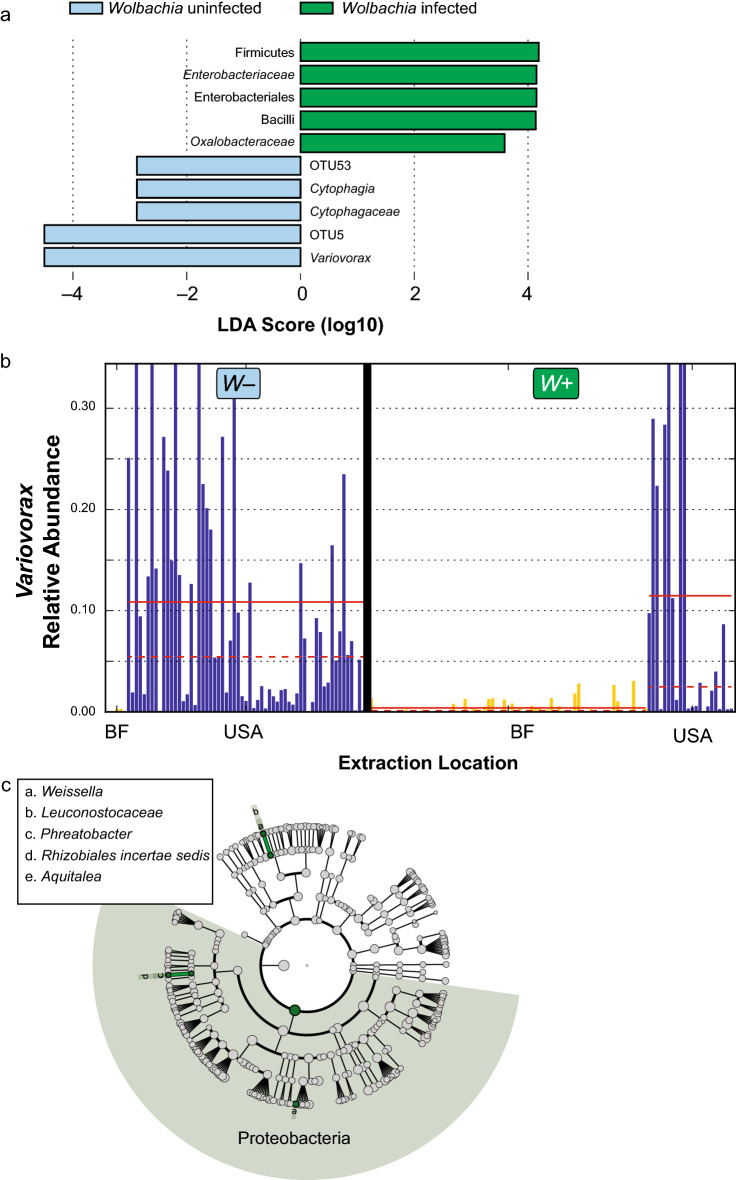
*w*Anga infection may alter specific OTU abundances. We investigated potential OTUs that correlated with *w*Anga infection status using the tool LEfSe^31^. We observed many OTUs and their upper levels of taxonomies associated with *w*Anga, both infected (green) and uninfected (light blue). Here we plot their log_10_ LDA score for the top 5 scores from both infected and uninfected mosquitoes (**a**). Using the extraction location as a sub-class, we observed an unbalanced number of *w*Anga-positive samples from the samples that were extracted in Burkina Faso (BF, yellow) compared to the USA (blue). Here we plot *Variovorax* relative abundance in each sample, separated by both *w*Anga infection status and extraction location (**b**). *Variovorax* is no longer robustly correlated with *w*Anga uninfected samples as there were a very limited number of mosquitoes extracted in BF that were uninfected with *w*Anga. Upon reanalysing mosquitoes that were extracted only in the USA, we observed a number of OTUs and taxonomies positively associated with *w*Anga infection, including Proteobacteria as a whole. Here, we display the hierarchical tree based on taxonomy of these associated taxa highlighted in green (**c**). All figure panels were generated using LEfSe^31^.

We sought to disentangle potential OTUs (i.e., individual bacterial species) that correlate with *w*Anga infection status, given that infection status is also correlated with several other biological and technical sample attributes, using multiple orthogonal approaches. The first approach was to use LDA Effect Size (LEfSe), which can find continuous features (i.e., taxonomy abundances) that vary based on categorical variables (e.g., *w*Anga infection status)^31^. Using LEfSe, we found multiple OTUs and associated taxonomies that differed between *w*Anga infection status (Fig. 4a). The strongest signal was an OTU assigned to the *Variovorax* genus, which was highly and significantly negatively associated with *w*Anga infection (p = 2.96e−11, logLDA = 4.5), with the majority of *w*Anga-positive mosquitoes containing little to no *Variovorax*, and the majority of *w*Anga-negative mosquitoes containing a high abundance of this OTU (Fig. 4b). *Variovorax* are Beta-Proteobacteria within the *Comamonadaceae* family, are gram-negative, and are found ubiquitously through many environments, including soil, plant rhizosphere, and many aquatic environments^32–35^. The most well-known member of the genus is the type species *V. paradoxus*, which has been extensively found in heavily-polluted environments as it can degrade a wide array of organic pollutants^36^. *Variovorax* has also been previously isolated from anopheline mosquitoes^37^.

LEfSe also takes a sub-class into account, which tests if the main class results are consistent between sub-classes (e.g., *w*Anga infection status compared across both extraction locations). However, when we opted to use extraction location as a sub-class, our power was significantly reduced due to having only six (out of 69) *w*Anga-negative mosquitoes extracted in Burkina Faso. Thus, using this approach, we did not have the power to statistically determine if our previous results were robust to different extraction locations.

We therefore took two alternative approaches to circumvent the possible confounding effect of extraction location. First, we used LEfSe to analyse only the subset of samples that were extracted in the USA, and excluding samples that were extracted in Burkina Faso. With the caveat that this dataset was unbalanced (with more mosquitoes that were *w*Anga negative than *w*Anga positive), we found four unique taxa that were all positively associated with *w*Anga infection (Fig. 4c). These taxa included the phylum Proteobacteria (p < 0.05, logLDA = 4.5), as well as three OTUs assigned to the genera *Aquitalea*, *Phreatobacter*, and *Weissella* (all p < 0.05, logLDA > 2). *Aquitalea* has been previously isolated from aquatic environments^38,39^. *Phreatobacter* has been isolated from water used for industrial purposes^40^. *Weissella* is a Firmicute that has been isolated mainly from fermentable foods, but some *Weissella* species have been found in the guts of various insects^41,42^. *Aquitalea* and *Phreatobacter* are within the phylum Proteobacteria, though the majority of OTUs within Proteobacteria were not significantly different (Fig. 4c). Proteobacteria levels appeared to be driven by certain non-infected mosquito samples that had much lower levels of Proteobacteria as a whole (Supplementary Fig. 4a).

Next, we employed MaAsLin, which uses multi-linear regression to take into account multiple sample variables in one model^43^. Many OTUs were associated with alive vs. dead at time of collection (n = 27), collection day (n = 107), and extraction location (n = 39) (Supplementary Table 1). Three OTUs were associated with *w*Anga infection status: OTU150 (*Sphingomonas*) and OTU232 (Bacteria) were negatively associated, while OTU271 (*Bacillus*) was positively associated (Supplementary Fig. 4b). To note, OTU232 and OTU271 were also concordant in the original LEfSe result including all samples, but OTU150 was not found. *Sphingomonas* are Alphaproteobacteria and have been known to have a wide array of biosynthetic and biodegradative properties and occasionally cause nosocomial infections in humans^44,45^, whereas *Bacillus* are ubiquitous gram-positive species found within the Firmicutes phylum, commonly found in soil and other natural habitats^45^.

As we discovered subsequent to sample collection for this study, *w*Anga influences *Anopheles* oviposition timing^16^, and therefore collection day in this experiment. We therefore opted to exclude the sample variable ‘day’ from MaAsLin analysis due to a real biological mechanism masking potential smaller, *bona fide* correlations^16^. Upon re-performing analysis, we found a different set of OTUs associated with *w*Anga infection status (Supplementary Table 2). OTU150 (*Sphingomonas*) again was negatively correlated with *w*Anga infection, while OTU148 (*Pseudomonas*), OTU196 (*Lysinibacillus*), and OTU2500 (*Janthinobacterium*) were all positively correlated with the presence of these bacteria (Supplementary Fig. 4b). To note, except for OTU150, these OTUs were concordantly found within the original LEfSe results as well. *Pseudomonas* has been previously reported as a member of the anopheline midgut microbiota^25,26,28,46^. One species of *Lysinibacillus*, *L. sphaericus* (previously *Bacillus sphaericus*), has been shown to have larvicidal properties against anophelines and has been used with some success as a pest control^47,48^. *Janthinobacterium lividum*, the type strain of the *Janthinobacterium* genus, is a gram-negative soil-dwelling beta-proteobacterium and has been shown to have antimicrobial properties^49^.

Previous reports have implicated *Asaia* as a competitive inhibitor of *Wolbachia* in *Anopheles stephensi*, where an individual mosquito can harbor either *Asaia* or *Wolbachia*, but not both at once^18,50^. We were able to detect one OTU assigned to the *Asaia* genus in 56 of 171 mosquitoes, with 28 (16%) mosquitoes having two or more counts. Contrary to previous results from these other reports, we found no negative correlation in our dataset between the abundance of *Asaia* and *w*Anga infection, as samples with higher levels of *Asaia* were evenly distributed between *w*Anga infected and uninfected mosquitoes (Supplementary Fig. 5).

## Discussion

There have been multiple studies of mosquitoes associating attributes of their microbiome with developmental life stage^25,26,29^, ecological (e.g., geographic, seasonal) differences^24,27,46,51^, insecticide resistance^52^, and resistance to infection by *Plasmodium* and other human pathogens^2–5,8–10,14,16,19,23,37,53–60^. There has been great interest and relevance for human health in the harnessing of the microbiome to block mosquito infection by human pathogens. In the *An. gambiae* species complex, several bacterial groups have been nominated for this task, including *Asaia*, *Serratia*, and the leading candidate *Wolbachia*^2,4,10,14–16,18,19,51,55,61^. To date, however, the relationship between *Wolbachia* and the rest of the microbiome of *An. gambiae* species complex has remained unstudied.

Here, using 16S rRNA sequencing to investigate the microbiome of wild-caught, blood-fed *An. coluzzii* female mosquitoes in Burkina Faso, we found microbiota in *An. coluzzii* similar to those described in previous studies and to the microbiome of other *Anopheles* mosquitoes, with a predominance of Proteobacteria and a smaller representation of other phyla^25,28,62^. At the family level, we also observed several previously reported families^24,27,28,30,46,50–52^, including a large fraction of *Comamonadaceae* and *Moraxellaceae*, *Pseudomonadaceae*, and rarely *Enterobacteriaceae*. We also found a large amount of variation between individual microbiomes at each of these levels, as previously observed^25,27–29,46^. Our samples had been previously divided into *w*Anga-positive or *w*Anga-negative status (using a *Wolbachia*-specific, highly sensitive PCR assay (Fig. 1)^16,63^. In our 16S rRNA sequence data, we were however able to detect sequence assigned to the *Wolbachia* genus in only one mosquito, which was likely due to a combination of limited sequencing depth and low infection intensity and is concordant with other anopheline microbiome studies^23–25,27,28,46,51^.

We investigated the potential interactions between *w*Anga infection and the *An. coluzzii* microbiota composition. We found no major differences between *w*Anga-infected vs. non- infected mosquitoes, as shown by both alpha and beta diversity measurements. This is in agreement with a previous report of the influence of *Wolbachia* infection in the related mosquito *Aedes aegypti*, where similar phylum-level profiles were observed irrespective of the presence of *Wolbachia*^13^. Upon closer investigation of finer-level details of the microbiota, we observed several OTUs and bacterial taxa that strongly correlated with *w*Anga infection status, including the strongest signal, an OTU assigned to the genus *Variovorax*. These results may indicate that these bacterial taxa may be promoting or disrupting the ability of *w*Anga to colonize the mosquito host, depending on whether they are positively or negatively correlated with *w*Anga infection, respectively. Alternatively, the presence of *w*Anga itself may instead influence the ability of these other microbes to colonize the mosquito, through either direct competition or potential stimulation of the host immune system^13,58,60^. However, due to the impact of *w*Anga infection on oviposition timing^16^ and the use of female mosquitoes collected following oviposition in this study, we cannot completely disentangle the potential for batch effects from *bona fide* signals. However, we feel that batch effects are unlikely to explain the observed OTU associations with *Wolbachia*, given that these mosquito samples had significant bacterial biomass and may therefore be less susceptible to contamination during DNA extraction^64^. More sophisticated bioinformatics approaches that attempt to account for stratification yielded several additional candidate OTUs associated with *w*Anga infection, motivating future studies employing randomized sample processing.

From this study, we predict that if a native *w*Anga strain were to be used for future vector control efforts the overall microbiota of the mosquito would be unlikely to change. Specific low-abundance bacterial taxa, such as *Variovorax* or *Asaia* may act as competitive inhibitors of *Wolbachia* infection^18,50^, and thus, could be impediments to *Wolbachia*-based disease transmission efforts in the absence of a strong CI phenotype. More studies are needed to validate our findings and to further explore how the full diversity of the *An. coluzzii* microbiome could be exploited for vector control.

## Conclusion

Our previous findings that *Wolbachia* imparts phenotypic effects on *An. coluzzii* mosquitoes as well as *P. falciparum* parasites appear to not be driven by or associated with changes in the overall composition of the microbiota of the mosquito. We observe differences in the abundance of select species and taxa within the microbiota, though these differences are difficult to disentangle from potential batch effects deriving from the impact of *Wolbachia* infection on oviposition timing^16^. Future work using both laboratory and natural populations of *An. coluzzii* will further elucidate possible microbe-microbe interactions between *w*Anga and other members of the resident microbiota, and may inform future efforts to control *Plasmodium* malaria parasite transmission.

## Materials and methods

### Collection of samples and DNA extraction

*Anopheles coluzzii* were previously collected from the village of VK5 (11°23′N; 4°24′W) in the Vallée du Kou, 30 km northwest of Bobo-Dioulasso in Burkina Faso in September 2014^16^. Blood-fed adult females were captured inside houses within the village, through a longstanding collaboration and with informed consent of the house owners, and maintained in an insectary on a 5% glucose diet for up to 3 days for observation. They were sacrificed immediately upon oviposition. The head was separated from the carcass, as insect heads have reported to contain PCR inhibitors that can give false negative results^65,66^, while the headless carcass was used for downstream DNA extraction. Though *Wolbachia* infections localize to reproductive organs, we profiled the whole (headless) carcass to explore potential impacts on other organs, including the midgut. Mosquitoes collected on day 1 (n = 29) and day 2 (n = 56) were extracted for DNA sequencing in Burkina Faso using the Qiagen Blood and Tissue kit as previously described^16^. The rest (n = 86) from day 1 (n = 21), day 2 (n = 22), and day 3 (n = 43) were stored in RNAlater (Thermo Fisher Scientific) and extracted using the Qiagen Blood and Tissue kit at a later date in the USA. This extracted DNA was used to assess *Wolbachia* via 16S, as previously described^16,63^. In brief, detection of *w*Anga in mosquito carcasses was performed by nested PCR amplification of the 16S rDNA region using *Wolbachia*-specific primers (W-Specf: 5′-CATACCTATTCGAAGGGATAG-3′, W-Specr: 5′-AGCTTCGAGTGAAACCAATTC-3′) and specific internal primers (16SNF: 5′-GAAGGGATAGGGTCGGTTCG-3′, 16SNR: 5′-CAATTCCCATGGCGTGACG-3′). For positive samples, the sequence of the resulting 412 bp fragment was determined by Sanger sequencing, and then both *w*Anga-infected and uninfected samples were submitted for 16S rRNA sequencing. Mosquito species was determined using PCR amplification of the extracted DNA with the S200 X6.1 locus (forward 5′-TCGCCTTAGACCTTGCGTTA-3′, reverse 5′-CGCTTCAAGAATTCGAGATAC-3′)^67^.

### 16S rRNA sequencing and analysis

#### Sequencing

Extracted DNA from mosquitoes was used to construct 16S rRNA sequencing libraries targeting ~ 250 bp in the V4 hypervariable region using 515F (5′-GTGCCAGCMGCCGCGGTAA-3′) and 806R (5′-GGACTACHVGGGTWTCTAAT-3′) PCR primers^68^. Then, libraries were sequenced using an Illumina MiSeq with paired end reads of 175 bp in length. Paired FASTQ files were generated and used in downstream analysis described below.

#### OTU table generation

A combination of QIIME (v1.4)^69,70^ and the UPARSE pipeline within the program usearch (v8.1.1861 64-bit)^71,72^ were used to generate Operational Taxonomic Units (OTUs) from the 16S rRNA sequences. In brief, using QIIME, paired reads from the V4 region were merged into one overlapping sequence for each read pair. Chimeric sequences were detected and filtered out. Then, unique sequences within the data were generated using usearch derep_fulllength. OTU clusters were generated from these unique sequences using usearch cluster_otus with a minsize of two, discarding singletons. Taxonomy was assigned to the OTU clusters using the utax algorithm within usearch with the RDP v16 database provided by the usearch author. Finally, an OTU table, tabulating read counts per OTU per sample, was created using usearch_global with an identity of 97% (-id 0.97).

#### Diversity analysis

The feature table^73^ and diversity modules from QIIME 2™ (version 2017.12.1 from qiime2.org)^70,74^ was used to filter and analyse the OTU table created above. First, the OTU table was filtered to remove OTUs present in only one sample, as well as present in fewer than 50 total counts across all 171 samples. This reduced the table from containing 3,189 to 916 OTUs, removing rare and ultra-low abundant OTUs.

This reduced table was then used as input for alpha rarefaction analysis with QIIME 2™, generating Chao1 measurements of down-sampled OTU tables from 1,000 to 20,000 counts per sample. Using this rarefaction curve, we determined 10,000 counts per sample was sufficient to measure the diversity within the majority of samples while retaining the majority of samples.

We thus down-sampled the OTU table to 10,000 counts per sample, which retained 11.6% of all counts and 144 (84%) samples, as samples containing fewer than 10,000 counts were excluded from the filtered table.

This filtered even-depth table was used to generate alpha diversity measurements (Chao1^75^) as well as beta diversity measurements (Bray Curtis^76^) using QIIME 2™. The Kruskal–Wallis test was used to compare Chao1 values for different groups of samples (e.g., *w*Anga infected vs uninfected). Bray Curtis measurements were input for Principal Coordinate Analysis to visualize how similar pairwise samples were to each other in two dimensions using EMPeror^77,78^. In addition, PERMANOVA with 10,000 permutations was used to assess how similar groups were to each other using Bray Curtis distances.

#### OTU-level analysis

LDA Effect Size (LEfSe)^31^ was used to assess correlations between OTUs and higher levels of taxonomy with various phenotypes (e.g., *w*Anga infection status, day collected, mortality [i.e., collected alive or dead], DNA extraction location). Input for LEfSe was the downsampled to 10,000 count, rare OTU filtered OTU table. The Galaxy implementation from the Huttenhower group was used (https://huttenhower.sph.harvard.edu/galaxy/). Default parameters were used.

The Huttenhower group Galaxy implementation of Multivariate Association with Linear Models (MaAsLin)^43^ was used to investigate correlations of individual OTUs with multiple sample metadata at once, including those described above. Again, input for the tool was the downsampled, filtered OTU table. Default parameters were used.

### Other statistics and graphing

All other statistics and plotting were performed in R (v3.4.3)^79^ using RStudio (v1.1.423)^80^.

## Supplementary information

Supplementary Figure Legends.

Supplementary Figure S1.

Supplementary Figure S2.

Supplementary Figure S3.

Supplementary Figure S4.

Supplementary Figure S5.

Supplementary Table S1.

Supplementary Table S2.

## Data Availability

All raw sequence data from this work can be found at NCBI under BioProject PRJNA294068. Only a subset of samples from the BioProject were used in the analysis.
