# Supplementary material for: The Anopheles coluzzii microbiome and its interaction with the intracellular parasite Wolbachia
Source: Sci Rep. 2020 Aug 14;10:13847. doi: 10.1038/s41598-020-70745-0 (PMC7427791; doi:10.1038/s41598-020-70745-0)
Supplement: Supplementary file 1 — Supplementary Figure Legends. [file 41598_2020_70745_MOESM1_ESM.docx]

**Supplementary Figure 1. 16S rRNA sequencing metrics** The majority of samples contained more than 10,000 reads (a). The relative abundance of the four “core” OTUs observed in all 144 mosquito samples with a minimum of 10,000 reads (b). Alpha rarefaction analysis using the Chao1 metric indicates that we have observed the majority of diversity using a minimum of 10,000 reads per sample (c).

**Supplementary Figure 2. Alpha diversity between different categories.** Alpha diversity was not significantly different between mortality (*i.e.*, collected alive vs dead) (a) or samples that were extracted in Burkina Faso vs the US (b). However, alpha diversity was higher in mosquitoes that were collected on the first day vs the second or third day (c).

**Supplementary Figure 3. *w*Anga infection status correlates with other metadata** *w*Anga infection status correlated both with extraction location (a) and day mosquito was collected (b), even after p-value correction. Infection status also correlated with whether mosquitoes were alive or dead at time of collection, but was not significant after FDR correction (c). To note, due to batch processing of mosquitoes as they were sacrificed, the majority of mosquitoes from day 2 and to a lesser extent day 1 were extracted in Burkina Faso, and all mosquitoes collected on day 3 were extracted in the USA (d), stratifying *w*Anga infection with both biological and technical factors.

**Supplementary Figure 4. OTUs associated with *w*Anga infection**

The phylum *Proteobacteria* was increased in *w*Anga infected mosquitoes that were processed in the USA, though this trend may have been driven by a subset of *w*Anga negative mosquitoes that had abnormally low levels of Proteobacteria (a). Shown here is the fractional relative abundance of the phylum for each sample extracted in the USA. Dotted line indicates median and solid line indicates mean value for each category. Various OTUs were found to be associated with *w*Anga infection status through MaAsLin (b). Shown here are the relative abundances of each OTU across samples, separated by both *w*Anga infection status (left vs right) and extraction location (BF red and USA blue).

**Supplementary Figure 5.** ***Asaia* relative abundance is not significantly different between *w*Anga infected and uninfected mosquitoes.** We detected one OTU assigned to the *Asaia* genus without our dataset. This OTU was not significantly different in abundance between *w*Anga negative mosquitoes (left) and *w*Anga positive mosquitoes (right), though in fact there was a slight elevation of the mean abundance in those infected with *w*Anga (horizontal solid lines), though the median abundance was zero for both groups, as *Asaia* was below the limit of detection for the majority of samples.
